# Dynamical modules in metabolism, cell and developmental biology

**DOI:** 10.1098/rsfs.2021.0011

**Published:** 2021-04-16

**Authors:** Johannes Jaeger, Nick Monk

**Affiliations:** ^1^ Complexity Science Hub (CSH) Vienna, Josefstädter Strasse 39, 1080 Vienna, Austria; ^2^ School of Mathematics and Statistics, University of Sheffield, Hicks Building, Sheffield S3 7RH, UK

**Keywords:** modularity, dynamical systems, activity-function, regulatory networks, metabolic control, systems biology

## Abstract

Modularity is an essential feature of any adaptive complex system. Phenotypic traits are modules in the sense that they have a distinguishable structure or function, which can vary (quasi-)independently from its context. Since all phenotypic traits are the product of some underlying regulatory dynamics, the generative processes that constitute the genotype–phenotype map must also be functionally modular. Traditionally, modular processes have been identified as structural modules in regulatory networks. However, structure only constrains, but does not determine, the dynamics of a process. Here, we propose an alternative approach that decomposes the behaviour of a complex regulatory system into elementary activity-functions. Modular activities can occur in networks that show no structural modularity, making dynamical modularity more widely applicable than structural decomposition. Furthermore, the behaviour of a regulatory system closely mirrors its functional contribution to the outcome of a process, which makes dynamical modularity particularly suited for functional decomposition. We illustrate our approach with numerous examples from the study of metabolism, cellular processes, as well as development and pattern formation. We argue that dynamical modules provide a shared conceptual foundation for developmental and evolutionary biology, and serve as the foundation for a new account of process homology, which is presented in a separate contribution by DiFrisco and Jaeger to this focus issue.

## 1. Introduction: the modular epigenotype

**Figure 1.**
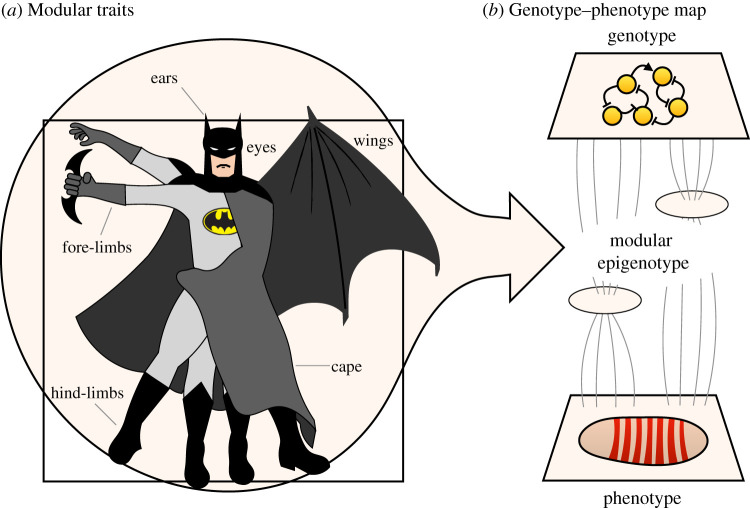
Modularity of the genotype–phenotype map. (*a*) Phenotypic traits are modular. They are distinguishable in terms of either structure or function, and are able to evolve in quasi-independent ways. An example is the specialization of vertebrate fore-limbs into arms and wings. (*b*) This implies that the processes that generate these traits—the epigenotype of the organism, which mediates the mapping from genotype to phenotype—must also be modular and dissociable (at least to some extent). Adapted from Jaeger & Monk [1]. See text for details.

Organisms are characterized by distinguishable metabolic, physiological, developmental, morphological and behavioural traits. These phenotypic traits are modular, able to evolve in a quasi-independent manner (figure 1*a* [1]) (see [2,3]). Simon [4,5] was among the first to point out that such ‘near-decomposability’ must be a fundamental and universal property of adaptive complex systems. Similar ideas are implicit in Stuart Kauffman's notion of biological systems being poised on ‘the edge of chaos’—with modular ‘islands of chaos’ among a large percolating component of ‘order’ [6] (see [1] for a detailed discussion). By limiting the off-target (pleiotropic) effects of mutations, a modular architecture enables evolution through targeted selection for specific trait functions. Wings are adapted for flying, and legs for walking, because fore- and hind-limbs in aerial vertebrates can evolve quasi-independently: even though they share a common underlying structure, their shape and size can be altered in different directions (figure 1*a*). It is widely accepted today that *trait modularity* is a fundamental prerequisite for *evolvability*, defined as the capacity of a system to generate adaptive change [7–9]. Consistent with this, modular traits are ubiquitous in evolving organisms. They have been described at many different levels of organization, from behavioural patterns to morphological characters to intercellular signalling pathways to *cis-*regulatory sequences involved in gene regulation [10,11].

All biological traits are generated by some underlying regulatory dynamics [12–14]. These generative processes constitute the *epigenotype* of the organism [15–19], which is often represented as *a complex mapping from genotype to phenotype* (figure 1*b*) [20–22]. If we are to properly understand the evolutionary sources of phenotypic variability—the substrate on which natural selection can act—we must study the structure of this mapping in a mechanistic manner [1,12,13,23,24]. What we are ultimately interested in are the causal processes that generate the variational properties of an adaptive and evolvable complex system.

An important first step in this direction is to recognize that the quasi-independence of modular traits necessitates a modular structure of the underlying epigenotype (figure 1). The processes that generate traits must themselves be modular or *dissociable* (at least to some extent) for those traits to be able to vary quasi-independently [25]. Therefore, it should be possible to map the source of variation in specific traits back to variability in specific modular subsystems of the epigenotype. This is not a trivial task and the precise nature of epigenetic modularity remains elusive, which is the problem we tackle in this paper.

Elsewhere, we have developed a detailed theoretical account of dynamic modularity, which allows us to decompose the genotype–phenotype map into dissociable causal processes [1]. Here, we briefly reiterate the main features of our account, illustrating it with a range of examples. In the next section, we start with an overview of the different types of modules one can identify in biological systems, comparing the advantages and drawbacks of each kind of modular decomposition. We then continue by introducing our own dynamical and activity-based notion of modularity. The examples we provide represent regulatory processes from the fields of metabolism, cell and developmental biology. We conclude by discussing some of the wider implications of dynamical modularity, especially in evolutionary biology.

## 2. Kinds of modules

Modules, defined in the broadest sense, are parts, components or subsystems of a larger system that possess a specific functional or structural identity [26]. They must persist long enough to exert their effect [27]. Modules themselves can consist of different kinds of entities: physical objects or structures, regulatory pathways or processes, and even types of behaviour as we shall show here. Modularity in complex systems is always a matter of degree [8]. On the one hand, all modules interact with their context—usually in a structured and hierarchical way. On the other hand, a system is modular to the extent to which its components operate according to their own intrinsically determined principles [27]. In other words, modules exhibit internal causal cohesion coupled with a certain degree of autonomy from their context [28–30]. This means that modules can be re-used, since they are able to operate robustly across a certain range of circumstances [31].

**Figure 2.**
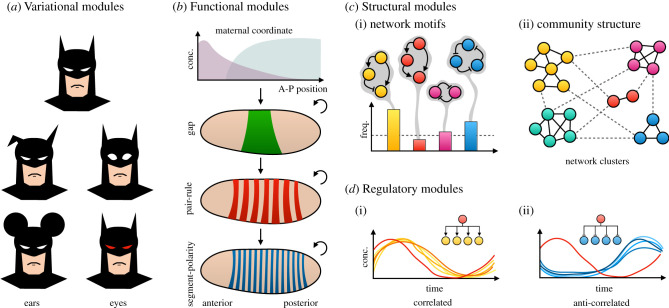
Different kinds of modules. (*a*) Variational modules are distinguished by their ability to vary quasi-independently of the rest of the system. In the example shown here, the shape, size and colour of ears and eyes can vary independently. These two morphological traits are, therefore, variational modules. (*b*) Functional modules are defined by the use-function their components contribute to. Shown here are the hierarchical layers of the *Drosophila* segmentation gene hierarchy. Genes in each layer cause similar kinds of phenotypes when mutated. They also show similarities in gene expression patterns (as shown here schematically). Downward arrows indicate hierarchical regulatory interactions; circular arrows indicate complex cross-regulation within each layer. (*c*) Structural motifs come in different flavours. Network motifs (i): small subnetworks that are statistically enriched above a given threshold (dashed line) compared to their expected occurrence in random graphs. Community structure (ii): modules correspond to clusters of network nodes (cliques) with denser connections among each other than to other modules. Often the definition of structural modules is more informal than those shown here. (*d*) Regulatory modules are a simple kind of dynamical module. They are defined by the correlated (i) or anti-correlated (ii) expression dynamics of their components, which indicate co-regulation by a mutual activator or repressor (see network insets). All of these classes of modules are reviewed in more detail in Jaeger & Monk [1].

There are many different kinds of modules, and various methods to distinguish them. One approach classifies modules according to their biological context, e.g. developmental versus evolutionary modules [27,32–36]. Another type of classification is based on what kind of entities a module is composed of: morphological modules consist of physical structures, developmental modules of processes [27,37]. Here, we will focus on how modularity is relative to the specific way in which a system is decomposed into parts or subsystems. Each such decomposition provides a different perspective on a complex system [38]. Decompositions based on functional, regulatory, structural or variational criteria result in modules with different properties and biological applications (figure 2) (see [1] for a more detailed discussion). We will briefly review each of these in turn, before we move on to modules that are defined in terms of their dynamical behaviour.

### 2.1. Variational modules

In an evolutionary context, modular traits are often identified based on their variational properties [8,39–41]. More precisely, variational modules are defined by a degree of statistical independence in the distribution of their properties compared to other modules. This kind of variational decoupling is what enables selection of specific traits for independent functions (figure 2*a*). As the flipside of the same coin, it also implies a significant overlap between variational and functional modularity (see below), since functionally related traits have a tendency to covary [39]. Variational modularity is a powerful concept underlying much of the discussion about homology [40] and the units of selection in evolutionary biology [2,3]. While it enables us to identify modules based on their observed variational properties, it fails to provide any causal-mechanistic explanations for the sources of this variability.

### 2.2. Functional modules

Another way to define a module is in terms of the function it performs. In contrast with the variational approach, this method presupposes that traits *have* separable functions [42]. Here, it is important to distinguish between different notions of biological function: we define the function of a trait not in terms of what it has been selected for in the past (selective-effect or aetiological function: wings are adapted for flying), but in terms of its contribution to the overall organisation and activity of the currently living organism (systemic or use-function: wings provide lift for aerial locomotion; [43,44]). Armed with this precise definition, we can now identify modules using interventionist (perturbatory) approaches such as genetics (see [45]; see also [24]). Once we have mutated a number of genes, we can classify them according to the specific use-function they contribute to. This is famously illustrated by the Nobel-prize-winning mutagenesis screen that characterized the segmentation gene network of the vinegar fly *Drosophila melanogaster* [46]. This screen yielded factors classified as gap, pair-rule, segment-polarity and homeotic genes, each contributing to a different kind of phenotype. Subsequently, it turned out that this functional classification also matches the regulatory layers in the segmentation gene hierarchy (figure 2*b*) [47,48]. This emphasizes the power of the functional approach in the context of genetic and molecular analyses in cell and developmental biology. While its ability to distinguish contributions to different use-functions is undisputed, this approach has severe limitations when it comes to reconstructing or ‘recomposing’ the detailed internal workings of a module. Perturbatory approaches such as genetics provide a good means of establishing parts lists of necessary module components. But apart from the simplest cases, they cannot provide a sufficient explanation of how these components interact to produce the orchestrated activity of the module [23,24]. To achieve this, we require more integrative approaches to modularity—based on structural, regulatory or dynamic criteria.

### 2.3. Structural modules

This approach is based on the idea that modules can be identified based on the local characteristics of connections within networks. There are several variations on this theme. Network motifs [49] are small subnetworks with a specific regulatory topology that are enriched in a given class of networks compared to their statistical occurrence in random graphs (figure 2*c*(i)). Another approach focuses on local community structure: it assumes that the density of connections within modules is higher than that of connections between them (figure 2*c*(ii)) [50–54]. In other words, modules represent ‘cliques’ of densely connected network nodes [55]. Such cliques can be disjoint, or can overlap with each other [56]. Mixed approaches to structural modularity combine network motifs with community structure [57], or employ a variety of empirical criteria (such as local topology, connectivity within the wider network and evolutionary conservation of nodes and interactions) to define different types of subnetworks and their hierarchical relations with each other more informally [58–61].

The strength of these structural approaches is that they can be readily applied to the abundant datasets on network structure that systems biology is currently generating. However, their basis in structure results in three significant limitations. (i) First, network structure constrains, but does not determine, function: even simple network topologies usually generate more than one type of dynamical behaviour [62–69]. In theory, this limitation can be overcome through a detailed quantitative characterization of network interactions. (ii) However, even in cases where this is practically possible, we are left with an additional issue: network behaviour is also exquisitely context-sensitive. Adding or subtracting a single connection in a subnetwork can completely change its repertoire of dynamic behaviours (see [68–70]). Dynamical behaviour depends not only on the structure, but also on the boundary conditions of a system. Because of this, structural modules do not really solve the problem of recomposition: apart from the simplest cases, static network structures cannot explain the orchestrated temporal activity of a module as a whole, in its particular regulatory context. (iii) On top of all this, it turns out that many multifunctional subnetworks do not show any structural modularity at all [71]. We discuss these problems in detail in Jaeger & Monk [1]. Suffice it to say at this point that structural approaches to modularity not only fail to solve the shortcomings of the functional approach, but also generate a range of problems of their own.

### 2.4. Regulatory modules

The difficulties with structural modularity can only be overcome by focusing directly on the activity of a module as a criterion for its definition. This introduces a third notion of biological function, the *activity-function* of a module, which is defined by what the module does or, more precisely, by the particular kind of dynamics it generates (see [72]; see also [14]). The simplest way of decomposing a process according to modular activities is to identify clusters of factors (e.g. gene products) that are expressed in a correlated or anti-correlated manner (figure 2*d*) [73]. Shared activating or repressive regulatory inputs can be used as an additional criterion for cluster identification (figure 2*d*, insets) [74,75]. Such co-expression clusters or synexpression groups are called regulatory modules [76–80] or activity motifs [55]. Regulatory decomposition overlaps with functional decomposition, since factors with (anti-)correlated expression patterns tend to contribute to the same use-function [74,75]. This approach is very widely used and scales well to whole-genome expression data. However, it is limited in two important ways. First, its predictions are of a probabilistic rather than mechanistic nature (although common regulators can give some mechanistic insight). In other words, it does not go beyond cataloguing clustered patterns to explain how they are generated. Second, and more importantly, regulatory decomposition only captures linear (anti-)correlations between expression patterns. The approach fails to detect activities that are based on more complex (especially feedback-driven) causal interactions. As we argue in Jaeger & Monk [1], these complex feedback-driven cases are often the most interesting and relevant examples of regulatory (and ultimately also functional) modularity.

In summary, none of the methods introduced above achieves the kind of causal-mechanistic decomposition of the genotype–phenotype map that we aim for. None of them fully captures the complex chain of nonlinear causal interactions that leads to the generation of a modular trait. Furthermore, combining methods does not help to overcome this limitation. What is needed instead is a novel approach, a true *decomposition under a process* (see [14]), which directly tracks the modular activities of the causal processes that constitute the dynamics of the whole.

## 3. Dynamical modularity

Variational, functional and structural approaches to modularity aim to identify concrete *modular objects* or *structures*: quasi-independent morphological characters, for example, or clustered subnetworks of interacting genes with a high internal cohesion and clearly defined boundaries towards the rest of the system. By contrast, the regulatory approach uses correlations in expression dynamics to decompose the complex *activity* of a system. It is its activity that defines the system as a process (see [14]). Seen from such a dynamical perspective, *a system is what it does*, not what it is composed of [1,12,13]. Accordingly, regulatory decomposition aims to identify and characterize the *subprocesses* that generate the full set of activities associated with a complex system. However, detecting linear correlations (as in the regulatory approach described above) captures only a small subset of all possible modular subprocesses. To cope with complex feedback-driven hierarchical regulatory dynamics, we need a more general strategy for decomposition under a process.

There is a second distinction to be highlighted: what we are looking for is a top-down method of dynamical decomposition that starts with the complex activities of *the whole system*. By contrast, most other approaches to modularity are bottom-up, decomposing a system into individual components underlying the generative process, or into elements of the outcome of the process. The whole point of functional and structural decomposition is to dissect a complex regulatory system into more manageable subsystems in the hope that the simpler activities of the resulting modules can be understood intuitively. The activity of the whole is then reconstructed by reassembling the isolated behaviours of individual modules. This is based on the brittle assumption that the activities of modules are independent of their wider network context, as they are in engineered systems that have been explicitly designed to have this feature. A top-down approach avoids this problem. It starts with the observable (usually complex) activities of the whole system, with the aim of *directly dissecting them into elementary activity-functions* [1,81].

With these two fundamental distinctions in mind, let us attempt to refine the definitions of dynamical modules (sometimes also called dynamical subsystems) that we have provided previously [1,70,81]. *A dynamical module or subsystem is defined by an elementary—distinguishable and quasi-autonomous—activity-function that corresponds to some dynamical regime within the broader dynamical repertoire of a complex system.* ‘Distinguishable and quasi-autonomous' means that the activity of a module must be characterized by recognizable dynamical properties that are invariant (at least qualitatively) across some range of background contexts (see also [14]). Once identified, these activity-functions can then be mapped onto specific structures, such as specific network components and their interactions. Importantly, these subnetworks need not be structurally modular. It is sufficient that they generate a distinguishable type of dynamics within the larger context of the system. Dynamical modularity lies within the activity of the system itself—not in the structure of the underlying network, which is secondary and may not be modular at all.

Let us now clarify and formalize what we mean by ‘elementary activity-function’, ‘dynamical regime’ and ‘dynamical repertoire’. The overall activity of a system corresponds to its orchestrated dynamical behaviour, typically described in terms of the time-dependent states of its components. For instance, the state of a gene regulatory network is determined by the concentrations of gene products (e.g. proteins or mRNA). States are related to each other by rules that formally define the interactions that constitute the system. In mechanistic models, these rules relate to the structure of the underlying causal processes [23,24]. Temporal sequences of related states represent the *trajectories* of the system. Different initial conditions result in different trajectories. The totality of trajectories over all possible initial conditions define the *phase portrait* of the system—the geometry of its *phase* or *state space*. This abstract space can contain various types of *attractors,* which are points or trajectories in the phase portrait to which other trajectories converge [82]. Converging trajectories make up the *basin of attraction* associated with an attractor. The type, quantity and geometrical arrangement of attractors and their basins define the *topology* of phase space. This topology determines the dynamical properties of the system: what it *can do* (the activities or dynamics it can exhibit) across a given range of initial conditions. Exploring the topology of phase space is central to the concept of dynamic modularity.

**Figure 3.**
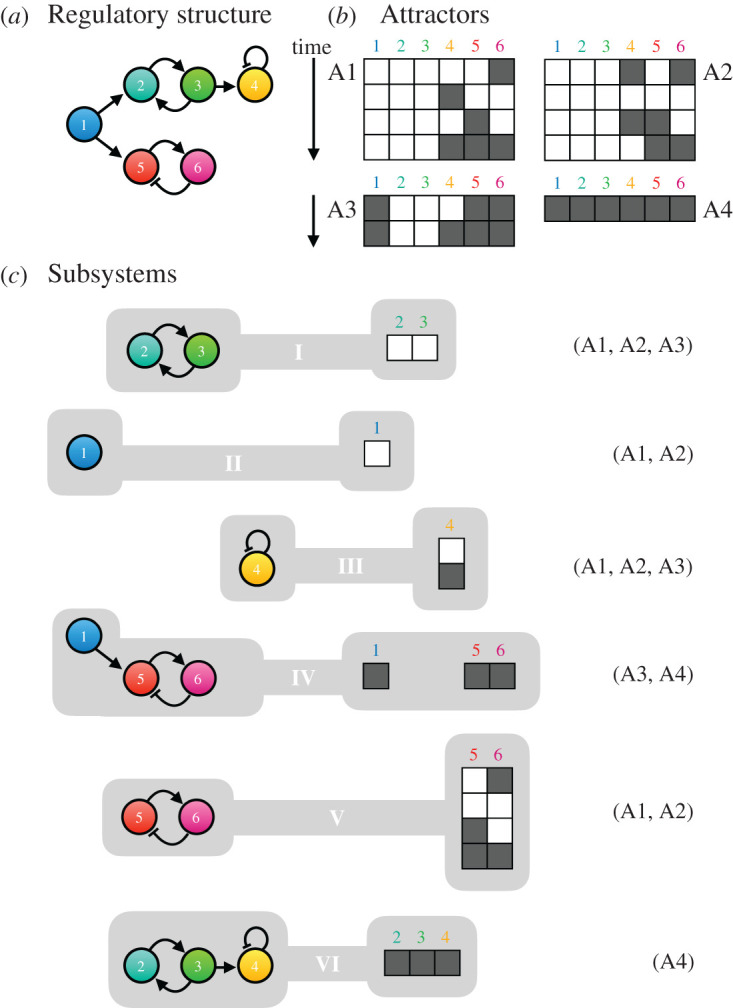
Dynamical modules in Boolean network models. (*a*) The regulatory structure of a simple Boolean network model. Arrows indicate activating interactions, T-bars represent repression. (*b*) The state space of this system contains four attractors, three of them repeatedly cycling through multiple states over time (A1–3), one of them stable (A4). Network components can be switched on (white) or off (dark grey). (*c*) Using the algorithm developed by Irons & Monk [81], this network can be subdivided into six dynamical modules or subsystems (indicated by roman numerals). Each implements a basic activity-function (characterized by the elementary subpatterns shown), which form parts of different attractors (as indicated by the terms in parentheses to the right). Together, these elementary activities add up to the more complex behaviour of the attractors. Each activity-function can be mapped back onto the structure of the network (shown to the left). Note that the subnetworks underlying each subsystem heavily overlap with each other, and show little discernible structural modularity in (*a*). Adapted from Irons & Monk [81].

In Boolean network models, with discrete on/off states and discrete-time behaviour, we can enumerate the totality of all possible trajectories and attractors in state space. This allows for an exhaustive algorithmic approach to the definition of dynamical subsystems. In brief, this approach relies on the identification of a set of primitive dynamic patterns among subsets of network components that combine to form the attractors (and hence the dynamical properties) of the system (figure 3) (see [81]; see also [1] for a more detailed review). These subsystems exhibit dynamical behaviour that is conserved in multiple different dynamical contexts (i.e. in multiple different attractors). This approach also establishes a hierarchy among subsystems by revealing how modules trigger one another in the attractors where they co-occur [81]. In this context, basic activity-functions can be precisely defined as the minimal dynamic ‘building blocks’ that are required to explain overall behaviour. This formal method of decomposition has been applied to Boolean models of the segment-polarity gene network in *Drosophila* [81], and of the yeast cell cycle [83] (see also §§5 and 6).

The rigorous algorithmic approach possible in Boolean models cannot be applied directly to systems with continuous-time dynamics and continuous states, such as those used to model processes with graded distributions of state variables (e.g. concentration gradients or smooth oscillations). Two main obstacles prevent generalization. First, it is not trivial to define adequate criteria to establish the similarity or equivalence of attractors when comparing continuous systems and, second, this poses a significant challenge when trying to map specific contributions of subsystems to a set of attractors present in the continuous phase space of a complex system. In figure 3, for example, it is straightforward to map subsystem dynamics back into both the attractors and the regulatory structure of the whole network. Since there are no discrete time steps, and the geometry of phase space differs in many subtle ways between subsystems and the whole model, this task is much harder to achieve in continuous systems. We will provide a concrete example of this in §6.

The situation is further complicated by the fact that the behaviour of continuous systems is often sensitive to the exact values of *system parameters* [82]. Such parameters can change under certain circumstances, albeit usually at much longer time scales than those of the dynamics of the state of the system. *Boundary conditions* are a particular kind of parameter that define the regulatory context and spatio-temporal domain in which a system exists. They can be altered by signalling inputs from outside the system, or by growth and other morphogenetic processes. Another example is parameters that determine the type and strength of regulatory interactions among components within a regulatory network. Genetic interactions are encoded by the genome, and yet they can be modified by developmental context—the presence of cofactors, tissue-specific or inductive signals, and environmental triggers—or genetic mutations, at an evolutionary time scale. Changes in parameter values affect the geometry of the phase portrait. One particularly drastic way in which this may happen is through *bifurcation* events, which cause changes in phase-space topology by altering the number and/or types of the attractors that are present [82]. States and parameters of a system together define its *configuration space*. Bifurcations occur at specific locations—called *bifurcation sets*—in this abstract space. These sets separate different *dynamical regimes,* which are characterized by specific topologies, i.e. specific qualitative arrangements of attractors and their basins relative to each other in phase space. During ontogenesis and phylogenesis, systems move along trajectories through their configuration space, traversing different dynamical regimes at different times of their existence. Each of these regimes represents a dynamical module.

The *dynamical repertoire* of a system is defined by those dynamical regimes that are present in the local region of configuration space that is currently accessible. This repertoire depends on internal system parameters (structure) as well as initial and boundary conditions (context). In less technical terms, the dynamical repertoire of a system determines the kinds of activities it can exhibit, given its particular circumstances and structural variations. In the following sections, we will present examples of dynamical modules that are activated in response to environmental triggers or intercellular signals, and modules whose behaviour depends on their position in an embryo, as well as on their relations to other modules. We will also dedicate some space to examples whose regulatory activities depend on tissue size and shape. All of this includes the possibility, of course, that a module will not get activated at all in a specific context. In that case, its activity will remain cryptic, an unexpressed potentiality. This has important implications for both physiology and evolutionary biology, which we will illustrate through examples in the following sections.

**Figure 4.**
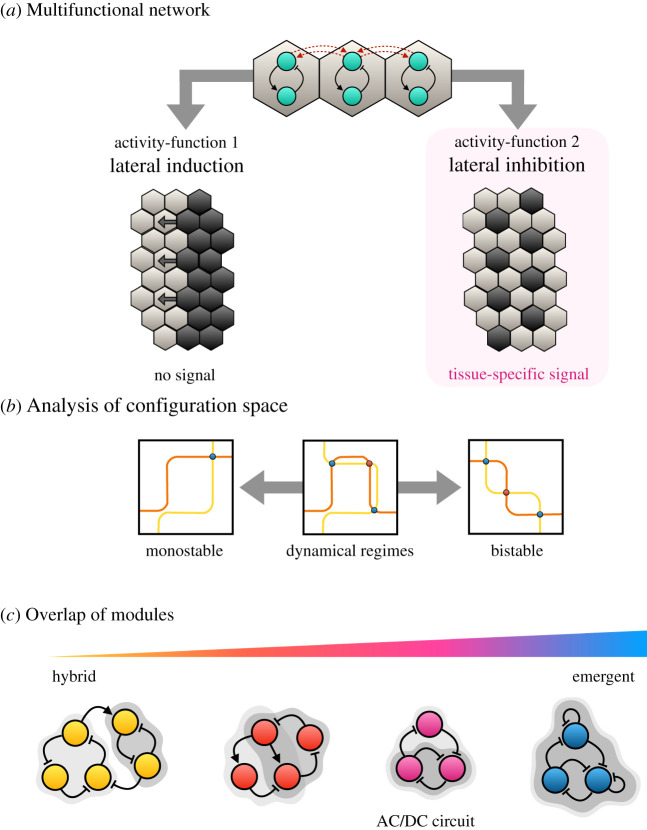
Dynamical modules in continuous network models. (*a*) The regulatory structure of a simple multicellular signalling network is shown on top. Black arrows indicate activating interactions; black T-bars represent repression. Dashed red arrows indicate intercellular signalling (activatory or inhibitory). This network can carry out two distinct activity-functions in the presence or absence of a tissue-specific modulatory signal. Left: during the process of lateral induction, uninduced neighbouring cells (light) are triggered to express a specific cell state (dark). This leads to a wave of gene induction sweeping across the tissue (indicated by black arrows). Right: during the process of lateral inhibition, signalling cells (dark) inhibit the expression of their cell state in neighbouring cells (light). This leads to a salt-and-pepper pattern of equally spaced signalling centres. (*b*) Analysis of configuration space reveals that activity-functions correspond to specific dynamical regimes, with transitions mediated by bifurcations between them. Example phase spaces are shown with nullclines (orange and yellow), attractors (blue dots) and saddle points (red dots) indicating distinct topologies of phase space. (*c*) Multifunctional networks differ in the extent of overlap between components that carry out different activity-functions (indicated by grey shaded backgrounds). Hybrid networks are structurally modular, with disjoint sets of components carrying out different activities (left). Emergent networks lack structural modularity: all their activities are carried out by the same set of components (right). Most multifunctional networks fall somewhere in between these two extremes. See §6, for the AC/DC circuit. Adapted from Jiménez *et al*. [71].

What we need to achieve then to identify dynamical modules in continuous systems is an analysis of the local geometry of configuration space, which aims to characterize the activities of a system in terms of the dynamical regimes that constitute its repertoire. Multifunctional networks are a particular case in point. They are defined by rich repertoires that contain multiple types of dynamical regimes, performing qualitatively different activity-functions in different contexts. Jiménez *et al*. [71] studied ensembles of simple multicellular network models that simulate lateral induction or lateral inhibition, depending on a contextual (tissue-specific) signal (figure 4*a*). These networks can thus be decomposed into (at least) two different dynamical modules—an inducing and an inhibitory one—which are actualized by the same network in different tissue contexts. The authors show that each activity-function is characterized by a specific topology of phase space, with stereotypical bifurcations between dynamical regimes (figure 4*b*). Depending on the regulatory structure of the model—the types and strengths of network interactions—these two activity-functions can be carried out by disjoint sets of network components (in *hybrid circuits*, where dynamical modules coincide with structural ones), or they can be carried out by the same nodes (in *emergent circuits*, where there is only dynamical, but no structural, modularity). Most multifunctional models fall on a spectrum somewhere between these two extremes, showing partial overlap between dynamical modules (figure 4*b*).

Complex nonlinear continuous system models present technical challenges not encountered in Boolean models. It is difficult to guarantee that all possible dynamical regimes have been captured, and the possibility that there is a more fundamental way to dynamically dissect the system cannot be excluded. Indeed, the configuration space of the system is always dependent on the context-dependent choice of variables and parameters we chose to formalize it. Pragmatically, we can start with observable activity-functions that are of interest to us, and characterize their dynamical properties in terms of their phase-space topology (plus the transitions between them in terms of bifurcations). We can then map their behaviour back onto the regulatory network. This provides a proof of principle that the analysis of configuration space can be used to identify and characterize dynamical models in continuous models (see §6). It also demonstrates that dynamical and structural modularity are dissociable, at least to some extent, and that many systems that show no structural modularity can still be dynamically modular, exhibiting multifunctional behaviour.

So far, we have provided a conceptual analysis of dynamical modularity. We have shown how it can be identified algorithmically in Boolean network models, and more pragmatically in models based on continuous formalisms. We now illustrate the practical potential of our account through a survey of real-world regulatory systems—metabolic, cellular and developmental—that exhibit dynamical modularity.

## 4. Modular metabolism

We first consider the notion of dynamical modularity in the context of metabolic systems. Metabolic flux is regulated at two different levels (see [84]). A first, immediate level of regulation is based on enzymes altering the rates of metabolic reactions, ‘lifting’ metabolic flux from the underlying network of thermodynamically possible reactions onto a separate time scale. A second, higher level of regulation changes the concentrations or activities of enzymes, thereby altering the fluxes and concentrations of intermediate metabolites (and the dynamic form—fixed, oscillatory, or chaotic) that characterize a steady state. This leads to several challenges for the traditional approach of subdividing metabolism into separate structural modules (classical metabolic pathways), with the aim of studying their behaviour in isolation. Such a structural approach relies on the assumption that the properties of the steady states that characterize a pathway's activity do not depend on its wider regulatory context. This is evidently not true.

To address this rather fundamental problem, it is useful to consider the metabolic network of the cell as a ‘molecular economy’, to be analysed in terms of supply and demand [84,85]. This approach fits perfectly with our notion of dynamical modularity, since it decomposes the complex activity of the metabolic network in terms of elementary activity-functions. These are defined through ‘blocks’ of metabolic reactions (metabolic subprocesses, really) that are linked through sets or cycles of intermediate metabolites. The flux as well as the concentrations of these intermediates depend on the generation of reactants by the supply block, and the consumption of products by the demand block. The use-function of such a system is to adaptively satisfy the demand for products, while maintaining homeostasis by keeping the concentrations of intermediate metabolites within a narrow range of concentrations. These seemingly contradictory requirements can be met if flux is largely controlled by demand, while homeostasis is maintained by the processes that supply the reactants [84]. A generalized supply–demand analysis can be applied to kinetic models of arbitrarily complex metabolic networks, with the aim of identifying and characterizing the conditions under which specific enzymes and metabolites really exert metabolic control [85]. For instance, it can be shown that allosteric feedback inhibition by the product of a biochemical pathway only works in a specific range of metabolite concentrations, which must lie far from equilibrium. In this sense, supply–demand analysis is a much broader and more robust tool for analysis than the more traditional structural approach to decompose metabolism, because the activity-functions of support–demand dyads are much more closely related to their use-function than the structure of a biochemical pathway.

This point is further emphasized if we focus more explicitly on the higher, regulatory level of metabolic control. There are two factors that particularly affect the function of a metabolic module, which explicitly depend on the dynamical properties of the system [55,86]. The first is *retroactivity,* defined as the extent to which the downstream regulatory effects induced by the upregulation of an enzyme's concentration or activity can alter its own activity-function. Retroactivity is basically a measure for how much feedback there is among the (dynamical) modules of a metabolic (or regulatory) system. One possible solution to this problem is a decomposition approach that minimizes retroactivity [86,87]. This approach led to the discovery of another dynamical characteristic important for modular regulation: *kinetic insulation*. Kinetic insulation minimizes retroactivity by separating the time scales at which different regulatory inputs (or perturbations) propagate through the system [55,86]. Its explicit dependence on reaction rates is obvious. All of this strongly implies that the identification of functional modules in metabolic systems *does* depend on kinetic properties, and requires a dynamical approach, especially since most metabolic processes, from bacteria to humans, are heavily feedback-driven.

## 5. Dynamical modules of the cell

So far, we have mainly discussed examples of modular systems that are spatially or functionally differentiated. Can we use our approach also for systems that are engaged in a seemingly unified temporal activity, such as the regulation of the eukaryotic cell cycle? Cell-cycle research is part of a broader investigation into biological oscillations, which also includes the study of circadian and other cellular and organismal rhythms [88–90]. The arguments we present here are generally applicable to this entire field.

All proliferative cells must progress through the stages of the cell cycle in a robust dynamical sequence. Since the cycle functions in a wide range of cell types and circumstances, the qualitative dynamics of the process must be robust and largely independent of context. At the same time, it must also be responsive to different inputs that regulate the rate and progression of the cycle. The cell cycle is, therefore, an excellent example of a dynamic module; it constitutes a fundamental modular component of the dynamics of a cell, which combines relative dynamical autonomy with tight integration into the system as a whole.

**Figure 5.**
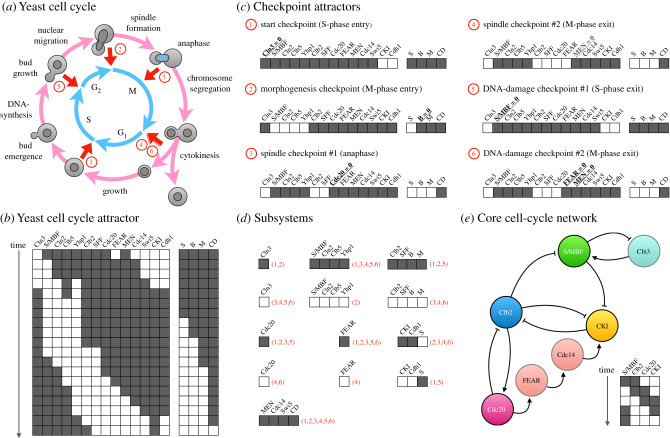
Dynamical modules of the yeast cell cycle. (*a*) The cell cycle of the budding yeast *S. cerevisiae.* Characteristic subprocesses are indicated on the outer circle (pink), cell-cycle phases on the inner circle (light blue). 1–6 demarcate cell-cycle checkpoints (in red). (*b*) The cyclical attractor shown in this panel is the only attractor in the state space of the Boolean cell-cycle model by Irons [83]. It accurately recapitulates the observed order of gene expression and other regulatory events observed in dividing yeast cells. The states in the block on the left represent expression states of cell-cycle regulators (on, white; off, dark grey). The states in the block to the right mark whether specific cell-cycle events, such as DNA synthesis (S), bud formation (B), mitotic entry (M) and exit (CD) have already occurred (event CD resets all the other events). This attractor can be considered as one integrated complex dynamical module in the wider regulatory context of the cell. (*c*) Checkpoint attractors: additional (fixed-point) attractors occur in the system if specific checkpoint regulators are fixed (as indicated in bold and underlined). They represent the regulatory states the cell gets arrested in, if it does not pass the checkpoint. These attractors can be subjected to dynamical decomposition (as in figure 3; see [81]), yielding the 13 subsystems shown in (*d*). The checkpoint attractors each subsystem occurs in are indicated between parentheses (in red). Several genes share the same states across subsystems. This means that the network can be collapsed into seven core components, which constitute the regulatory network shown in (*e*). In this core regulatory network, the nodes shown in red only propagate a state (controlling the timing of expression but not affecting attractor states). Similarly, the Cln3 node can be replaced by S/MBF auto-repression. This results in a minimal network with four nodes (S/MBF, Clb2, Cdc20 and CKI), which exhibits a single attractor that is qualitatively equivalent to the full cell-cycle attractor shown in (*b*). In this case, the dynamical decomposition of checkpoint regulation can be used to drastically reduce the complexity of cell-cycle regulation.

The components and regulatory structure of the eukaryotic cell cycle have been explored in detail, and form the basis of several models—some based on Boolean, some on continuous formalisms; some general, some specific to particular model organisms (reviewed in [91]). The molecular interaction networks can be represented as a number of linked regulatory modules (see [92,93]); here, we will focus on a Boolean model introduced by Irons [83] to perform a dynamical decomposition of the regulatory process underlying mitosis in the budding yeast *Saccharomyces cerevisiae* (figure 5*a*)*.* This study found the yeast cell-cycle system to be extremely robust, with all its transient behaviour converging onto a single cyclical attractor (figure 5*b*). In addition, a comparison between models with synchronous and asynchronous state updates yielded the surprising insight that cell-cycle control does not depend crucially on any fine-tuning of regulation or degradation rates. At first glance, therefore, the regulatory process underlying the yeast cell cycle seems to consist of one unified dynamical module represented by its single complex attractor.

However, a more detailed analysis reveals a type of temporal modularity that is based on the presence of checkpoints that occur at specific phases of the cell-cycle oscillation [94,95]. These checkpoints ensure that certain crucial processes have completed before the cell cycle transitions to a new phase. These are (1) the start checkpoint, which keeps cells from entering S-phase before sufficient growth has occurred, (2) the morphogenesis checkpoint, which allows entry into mitosis (M-phase) only if the bud is fully grown, (3,4) two spindle checkpoints, which prevent entry into anaphase or exit from mitosis if chromosomes are not properly aligned on the spindle, and (5,6) two DNA-damage checkpoints, which prevent exit from S- or M-phase in the presence of broken DNA strands (figure 5*a*). These checkpoints are controlled by specific genetic factors. If we block or eliminate the activity of these factors in the model, the system settles into a set of fixed-point attractors, which represent the arrested states of cells that have failed to pass the respective checkpoint (figure 5*c*). These checkpoint attractors can be subjected to dynamical decomposition (as in figure 3 [81]), which yields 13 distinct subsystems (figure 5*c*). These subsystems reveal that some components of the network are redundant, since they always occur in the same state across all subsystems. Thus, we can collapse the system into a network of seven core components, which define a simplified model of the core process underlying cell-cycle regulation (figure 5*e*). Moreover, three nodes in this core model provide only temporal control, but do not alter the attractor dynamics of the system. Their elimination yields a minimal core model with only four components, which exhibits a single cyclical attractor (figure 5*e*, inset) that is qualitatively equivalent to the more complex attractor of the whole system (figure 5*b*). This case powerfully illustrates how the dynamical decomposition of checkpoint regulation can be used to simplify and understand the complex dynamic mechanisms underlying cell-cycle regulation.

Another very promising field of application for dynamical modularity is cell–cell signalling. Conserved signalling pathways can be considered both structural and dynamical modules. Specific pathways are characterized by their canonical sets of ligands, receptors and downstream transduction factors, together with the stereotypical interactions that define them. In addition, non-canonical variants have been identified for virtually all of the major signalling pathways. This rich taxonomy can be further refined if we consider the fact that the effect of a signal crucially depends on its dynamics, duration, intensity, the state of the receiving cell (its competence) and whether the signal occurs in isolation or together with other signals (see, for example, [17,55,96–99]). By doing this, we move from a static view of signalling pathways as structural modules, to a processual view centred around their activity-functions and their complex regulatory context. Simply put, signalling activities are quintessential dynamical modules, and their analysis requires a dynamical approach.

**Figure 6.**
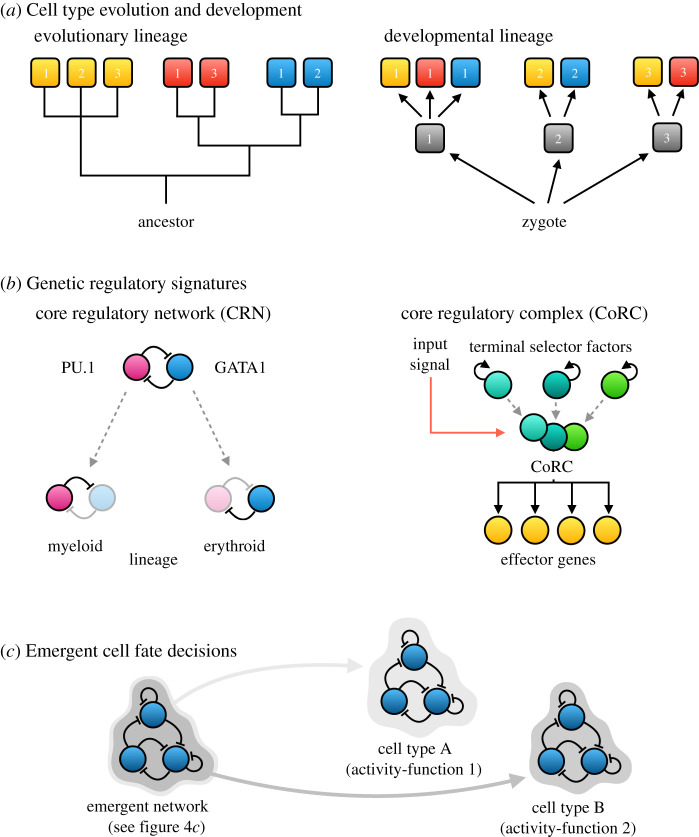
Cell differentiation. (*a*) Differentiated cell types can be considered equivalent to typical morphological traits, except that their evolutionary and developmental lineages often diverge. This figure shows an evolutionary and a developmental lineage tree for three different cell types (marked by yellow, red and blue) that occur in three different tissues (1–3). Tissue progenitors are shown in grey. (*b*) Genetic regulatory signatures: cell fate decisions in (*a*) are often assumed to require specific dedicated genetic circuitry. These can come in the form of double-negative (positive) feedback loops (core regulatory networks, CRNs; left), where either one of the components gets locked into expression in different cell lineages (e.g. PU.1 and GATA1 transcription factors in the myeloid–erythroid cell fate decision). An alternative hypothesis sees complexes of transcription factors (so-called terminal selectors) at the heart of cell type identity (core regulatory complexes, CoRCs; right). CoRCs often respond to external signals. Auto-activation provides the positive feedback to stably maintain cell fate. The assembled CoRC then activates a set of effector genes that determine the morphotype of the differentiated cell. (*c*) Cell-type-specific genetic signatures are sufficient, but not necessary, for cell differentiation. Alternatively, cell types can be defined through different activity-functions of an emergent gene regulatory network (figure 4*c*). In this case, the two cell types are *not* distinguished by any particular regulatory signatures, since the same circuit determines the fate of both.

Dynamical modules are also useful for considering cell differentiation. This example beautifully illustrates the difference between variational, functional, structural and dynamical approaches to modularity. In many ways, differentiated cell types are equivalent to classical morphological traits, even though their analysis is somewhat complicated by the fact that they tend to have divergent evolutionary and developmental lineages (figure 6*a*) [100,101]. Traditionally, cell types have been identified as *morphotypes*. For instance, different classes of photoreceptors can be recognized and classified by comparing their structure and the properties of their light-sensing use-function [100]. Thus, just like other morphological traits, cell types are functional modules. In addition, cell types are also modular in terms of their variational properties. Even closely related sister cell types in the same evolutionary lineage are able to vary quasi-independently, similar to morphological traits like fore- and hind-limbs (see §1). This calls for a mechanistic explanation of how they differ. Without such an explanation, the study of cell type evolution remains prone to misclassification, since structural and functional similarities could be due to convergent or parallel evolution rather than true homology (see [14]).

One hypothesis that has been put forward by multiple authors states that cell types must be characterized by specific genetic signatures. These signatures correspond to particular kinds of structural modules in the regulatory network of the cell (figure 6*b*). Graf & Enver [102], for example, identify them as *core regulatory networks* that consist of double-negative (positive) feedback loops between key regulators of cell differentiation, such as transcription factors PU.1 and GATA1, which are involved in the myeloid–erythroid cell lineage decision. The notion of *core regulatory complexes* (CoRCs [40,101]) is more refined: CoRCs are complexes of key regulators that lock into place their own state of expression by positive autoregulatory feedback, while activating target effectors (themselves also structural modules) that govern the actual differentiation process. Each cell type is thought to have its own characteristic CoRC—its own specific combination of regulators that are only activated together in one particular lineage. The functional modularity of cell types is thus reflected in the structural modularity of the whole-cell regulatory network [101].

For reasons outlined in §3, we find this structural approach too restrictive. While CoRCs may be sufficient, they are not necessary to explain the differences between cell types. Instead, cell differentiation could be driven by a single emergent regulatory circuit (*sensu* [71]; figure 4*c*), which implements different activity-functions in different developmental contexts through *the same* structural subnetwork (figure 6*c*). In this case, no unique genetic signature or regulatory complex is required to differentiate sister cell lineages, and it becomes necessary instead to find the *dynamical modules* that define a specific cell type. To the best of our knowledge, this has not yet been attempted, partly because this problem is not widely recognized, but partly also because identifying specific activity-functions among the enormous complexity of whole-cell dynamic behaviour is extremely difficult. One problem is that we do not yet have any detailed and rigorously validated dynamic models of whole-cell regulation. For this reason, it will be crucial to develop methods that can identify dynamical modules directly through analysis of time-series or condition-dependent gene expression data (see [55]).

The kind of argument we have presented here can be straightforwardly extended from CoRCs to *character identity networks* (ChINs), genetic signatures that are supposed to define the identity of morphological traits [39,40]. Again, the structural approach is too restrictive. ChINs may be sufficient, but are not necessary to explain the differences between traits, since morphological differentiation could be driven by a single emergent subnetwork implementing different activity-functions in different developmental contexts through the same network components and interactions. In this spirit, a revised account of *character identity mechanisms* (ChIMs) has recently been proposed that focuses more explicitly on the regulatory dynamics generating the properties of different morphological traits [103]. For more detailed discussions of this problem, see Jaeger & Monk [1] as well as DiFrisco & Jaeger [23,14].

## 6. Morphogenetic fields as dynamical developmental modules

Our discussion of morphological traits has already brought us into the realm of developmental regulatory systems. In this context, dynamical modules are closely associated with the central concept of classical embryology—the *morphogenetic field* [104]*.* Morphogenetic fields, as originally defined, denote a set of spatio-temporally localized interactions between regulatory processes, which generate a robust pattern during development (see [32]; see also [18,105,106]). Just like dynamical modules, morphogenetic fields represent developmental subsystems that generate distinguishable activity-functions (patterns), which remain invariant across some range of different contexts (robustness). In this sense, a dynamical module can simply be interpreted as a formalized representation of a morphogenetic field. Since dynamical modules also occur outside of developmental biology (as outlined in the previous two sections), morphogenetic fields can be thought of as constituting the subset of dynamical modules that apply to developmental processes. However, even though they represent a thoroughly processual view of development, morphogenetic fields emphasize the regulatory *structures* underlying pattern-forming processes [106], while dynamical modules focus more explicitly on the *activities* of a system. Also, for historical and methodological reasons, the boundaries of classic morphogenetic fields do not always coincide with those of the dynamical modules described here. Let us illustrate this point with a couple of examples involving the primary embryonic field in the vinegar fly, *D. melanogaster*.

The basic segmented body plan of *Drosophila* becomes determined at the blastoderm stage, during early development, before the onset of gastrulation. Historically, the whole blastoderm embryo has been treated as the primary embryonic field, which gets refined into more localized and organ-specific subfields as development proceeds [32]. Alternatively, this primary field itself can be subdivided into different subprocesses, contributing distinct activity-functions to the behaviour of the whole: nuclear division, migration and cellularization, axis and segment determination, dorsoventral and terminal patterning, and so on. Each one of these activity-functions constitutes a dynamical module. If we home in on the segment-determination module, we can discern the genetic hierarchy of maternal coordinate, gap, pair-rule and segment-polarity genes that generate a segmental molecular prepattern by the onset of gastrulation (figure 2*b*). Each layer in this hierarchy can be considered a dynamical module on its own. But we do not have to stop there. To arrive at the elementary activity-functions of the system, we need to dissect these subsystems even further.

**Figure 7.**
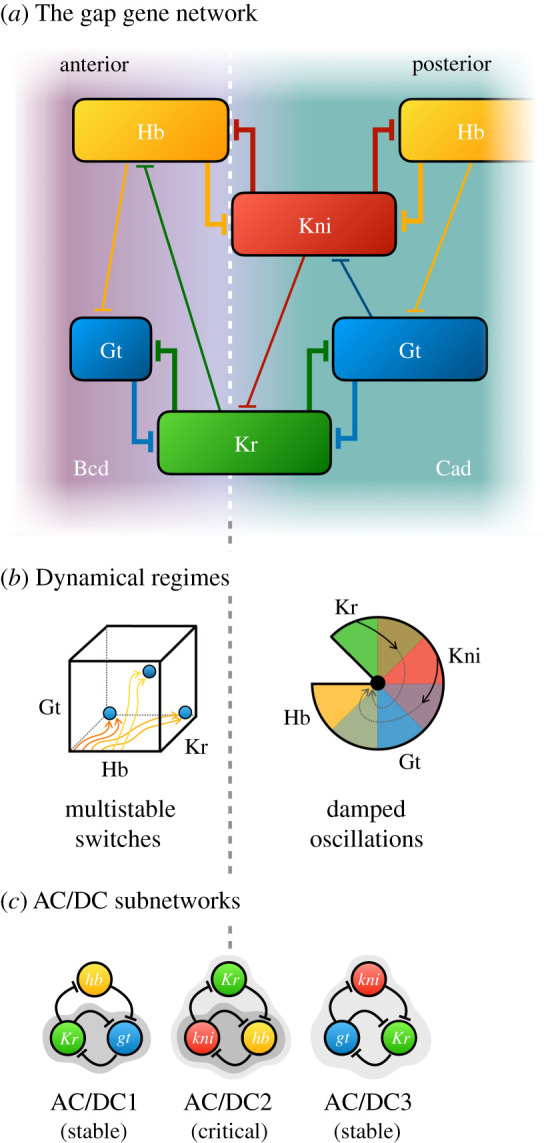
The *Drosophila* gap gene network*.* (*a*) A spatial diagram showing the relative positions of gap gene expression domains (coloured boxes) along the antero-posterior axis of the embryo. T-bars indicate repressive gap gene cross-regulation. Background colour indicates activating inputs by maternal gradients. (*b*) Dynamical regimes. In the anterior of the embryo, the stationary borders of gap domains are governed by switch-like multistable behaviour (schematically shown as a phase-space projection with different attractors in blue). The system approaches steady state over time. In the posterior of the embryo, shifting borders of gap domains are driven by an underlying damped oscillator, which causes a stereotypical succession of gap gene expression from *Kr* to *kni* to *gt* to *hb* in each blastoderm nucleus (as indicated by the colour wheel). Each nucleus starts its expression dynamics at a different position along the wheel, and the system remains far from steady state (as indicated by solid and dashed trajectories). This staggered succession of gene expression leads to the kinematic anterior shifts of the expression domains. The bifurcation boundary between the two dynamical regimes is indicated by a dashed vertical line. (*c*) AC/DC subnetworks: switch-like and oscillatory behaviours can be mapped back onto substructures of the gap gene network, each active in a different region of the embryo. These subnetworks heavily overlap in terms of their components, and all share the same network topology—that of the AC/DC circuit (cf. figure 8)—which is able to drive both switch-like and oscillatory behaviour (as indicated by dark and light grey shades). There is no one-to-one mapping in this system between dynamical regimes and individual subnetworks. Instead, AC/DC2 is in a state of criticality, exhibiting switch-like or oscillatory behaviour on either side of the bifurcation boundary in the middle of the embryo. Bcd, bicoid; Cad, caudal; Hb, hunchback; Kr, Krüppel; Kni, knirps; Gt, giant. Compiled from Crombach *et al*. [107] and Verd *et al*. [70,108].

As mentioned in §1, Irons & Monk [81] applied their algorithm for the detection of dynamical subsystems to a Boolean model of the segment-polarity gene network. Their analysis reveals a fundamental symmetry among the subsystems in this network, which explains its switch-like (bistable) overall activity (reviewed in detail in [1]). Similarly, Verd *et al*. [70] set out to identify dynamical modules in a continuous dynamical model of the gap gene system—a network that shows no discernible structural modularity (figure 7*a*). Earlier studies had discovered two distinct dynamical regimes for gap gene expression in the anterior and the posterior of the embryo [109–111]: while the expression boundaries of anterior gap domains are positioned in a stationary manner by switch-like (multistable) mechanisms, the borders of posterior domains shift towards the anterior over time, driven by damped oscillatory dynamics in the underlying system (figure 7*b*) [108]. These two different types of activities can be mapped onto three overlapping subnetworks of the system—each reproducing the patterning activity of the whole system in a distinct region of the embryo. Their compositional overlap means that these subnetworks are not structurally modular. Furthermore, all three share the same regulatory topology. They are AC/DC circuits (figure 7*c*) [112], able to produce both switch-like and oscillatory behaviour depending on the strength of their interactions and their regulatory context. While AC/DC1 (in the anterior) lies stably in a multistable regime, and AC/DC3 (in the posterior) exhibits damped oscillations, AC/DC2 (in the middle) straddles the bifurcation boundary at which the behaviour of the system changes. It is the only subcircuit that is in a state of criticality. Together, all three circuits reproduce the behaviour of the whole gap gene network. However, the dynamical regimes of the system do not map onto any unique and specific set of structural subnetworks. This means that the gap gene system lies closer to the emergent than the hybrid end of the spectrum of functional modularity (cf*.* figure 4) [71].

**Figure 8.**
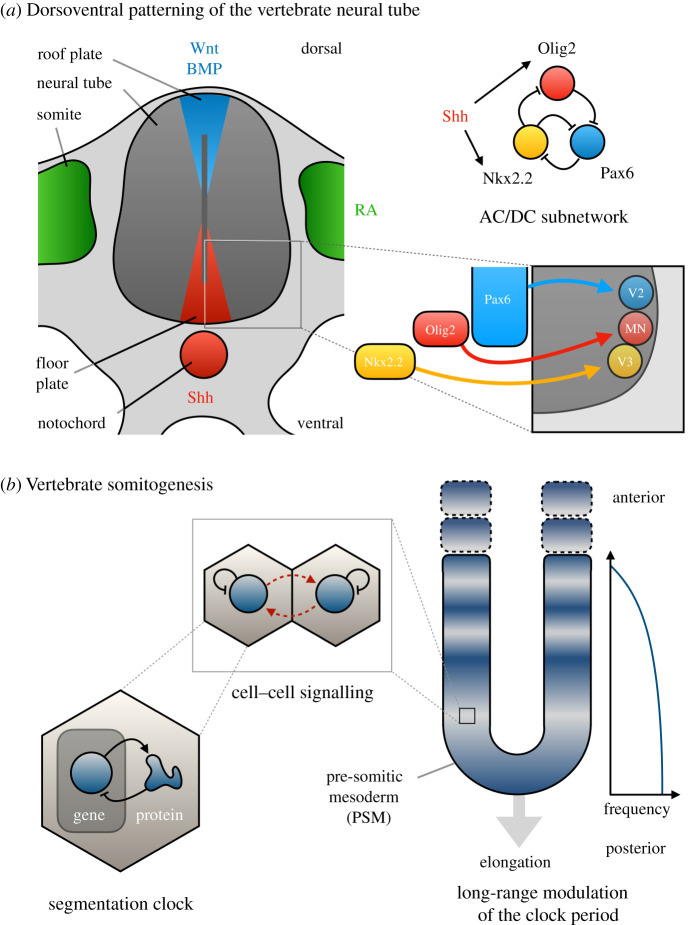
Dynamical modules in vertebrate pattern formation. (*a*) Dorsoventral patterning of the vertebrate neural tube. A cross-section through the developing neural tube and its neighbouring tissues is shown on the left. The tissue is patterned by morphogen gradients that emanate from the notochord and the floor plate (Sonic Hedgehog, Shh; red), from the somites (retinoic acid, RA; green) and from the roof plate (Wnt and bone morphogenetic proteins, BMP; blue). These gradients induce the expression of various target genes along the dorsoventral axis of the neural tube. On the bottom right, we show the ventral-most targets of Shh (Nkx2.2, yellow, and Olig2, red), as well as the repressor Pax6 (blue). Depending on the combination of target genes that are expressed in a given region of the neural tube, different populations of neural precursors are induced (V2, V3, MN). Top right: the interactions between the target genes form an AC/DC subnetwork (cf*.* figure 7*c*), which, in this case, is locked into a switch-like activity-function. Modified from Dessaud *et al*. [113] and Panovska-Griffiths *et al*. [112]. (*b*) Vertebrate somitogenesis: body segments form in a U-shaped elongating tissue called the pre-somitic mesoderm (PSM). Cells in this tissue exhibit oscillatory gene expression driven by a cell-autonomous segmentation clock, which is based on the auto-repression of Hes/Her transcription factors (left). Oscillations are synchronized by cell–cell signalling through the Notch/Delta pathway (middle). The period of these oscillations increases towards the anterior of the PSM, leading to kinematic waves of gene expression that travel through the tissue until they come to a stop and form a stationary molecular prepattern, which demarcates the future boundaries of the forming somites (indicated by dashed lines). These three activity-functions define the dynamical modules involved in somitogenesis. Modified from Oates *et al*. [114].

There are many more examples of dynamical modules in other species and developmental systems. The AC/DC subnetwork itself was originally described in the context of dorsoventral patterning in the vertebrate neural tube (figure 8*a*) [68,112]. This system shows many similarities to the gap genes: both subdivide a tissue into distinct territories of gene expression, and in both the interactions among target genes of a morphogen gradient are involved in setting shifting boundaries of gene expression [113,115,116]. As more empirical details about these interactions become known in the vertebrate neural tube, it will be interesting to subject them to dynamical decomposition. This opens the opportunity to better understand not only the temporal interpretation of positional information [117,118], but also the role of dynamical modularity in patterning precision [119,120].

Dynamical modularity is also important for the study of somitogenesis, the process by which body segments are established in vertebrate embryos. Unlike *Drosophila* segment determination, which occurs more or less simultaneously throughout the embryo*,* vertebrates form their somites—which are blocks of segmented mesodermal tissue—sequentially during the posterior extension of a tissue called the paraxial or pre-somitic mesoderm (PSM) (figure 8*b*) [114]. This process involves a regulatory network with three distinguishable dynamical modules (see also [14]): (i) cell-autonomous oscillations generated by a segmentation clock, (ii) synchronization of oscillations among neighbouring cells in the PSM through intercellular signalling, and (iii) a graded, long-range modulation of the clock period (the wavefront) causing it to slow down and eventually stop, creating a periodic spatial pattern that defines the position of somite boundaries, which form later in development (figure 8*b*) [114]. Again, the components of these modules overlap. There is no distinct structural modularity. For instance, factors involved in the Notch signalling cascade are involved in both cell-autonomous oscillations and their synchronization. The situation is less clear for the wavefront, which may be guided by long-range morphogen gradients, but there is at least one proposal that even this mechanism re-uses factors involved in the local dynamics of the oscillations [121]. Moreover, there is very little overlap among the sets of genes that oscillate in the PSM when comparing different vertebrate lineages, which indicates substantial evolutionary differences between the lineage-specific genetic mechanisms that implement the conserved activity-functions (see [122]; see also [14]). Finally, even the activity-functions themselves are tightly interlocked. Inhibiting cell–cell synchronization, for example, can alter the period of the cell-autonomous clock [123]. This does not invalidate analysis in terms of dynamical modules, however, as long as the activities of different models can still be distinguished from each other.

**Figure 9.**
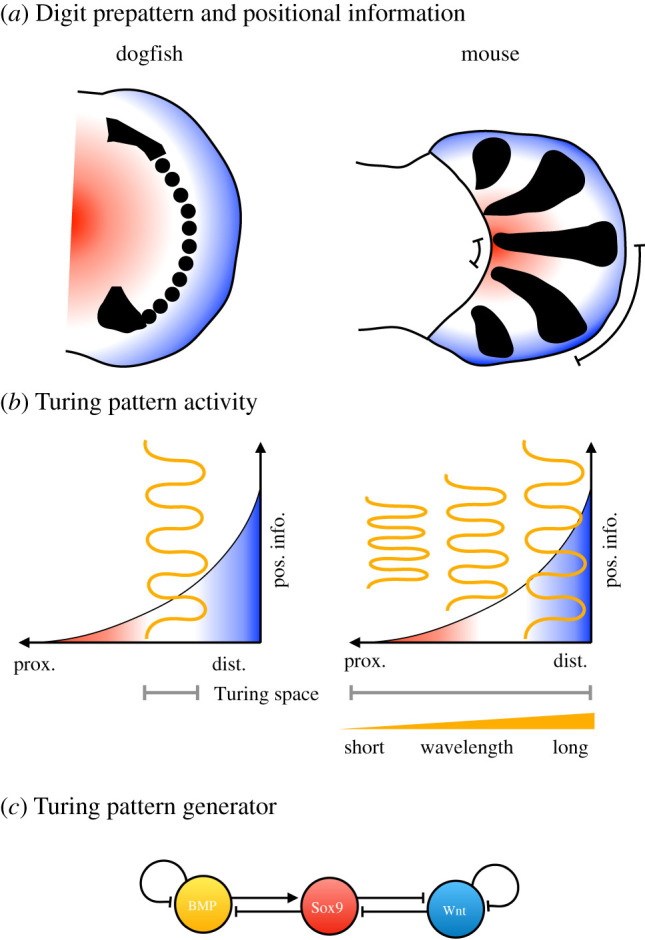
Turing patterning modules and the fin-to-limb transition. (*a*) A pectoral fin bud of a dogfish is shown schematically (to the left), and compared to a mouse limb bud at a comparable developmental stage (to the right). Black spots and stripes represent the expression of the Sox9 transcription factor, which forms a molecular prepattern for digit formation in mice. Red–white–blue gradients indicate graded positional information along the proximo-distal axis (provided by several different morphogen systems). (*b*) The pattern activity of the system responds differently to positional information in dogfish (left) and mouse (right). Turing space represents the region of configuration space in which the system can generate stable spatial patterns. In dogfish, these conditions only apply in the middle range (white) of the positional information gradient. In mice, Turing patterning occurs along the entire proximo-distal axis, but the wavelength of the output patterns increases towards the distal end. This leads to the splayed digit pattern observed in mouse limb buds. (*c*) Even though the patterning dynamics differ greatly between the two species, the underlying subnetwork generating the activities is highly conserved. Slightly modified from Onimaru *et al*. [130].

Turing patterning systems constitute another, very broad, category of dynamical modules [124–129]. They are invoked to explain diverse phenomena such as the formation of animal coat patterns, the spacing of hair and feathers on the skin of vertebrates, and the establishment of left–right asymmetry in bilaterian animals. Turing systems generally involve simple regulatory interactions among a small number of components—for instance, short-range activation combined with long-range inhibition. These simple mechanisms can generate a huge diversity of diffusion-driven patterns when implemented on tissues with various constant or growing shapes and sizes. Tissue context rather than network structure determines the outcome. One particular example that illustrates this point is the system that patterns the distal part of the vertebrate limb (the autopod). In tetrapods, such as mice, this process determines the position of the digits in the hands or feet (figure 9*a,b*, right) [131]. The autopod used to be considered an evolutionary novelty of land-dwelling tetrapods. However, recent experimental work in sharks identified an equivalent patterning system shaping the distal part of the chondrichthyan fin (figure 9*a,b*, left) [130]. The activity-function of this module is highly conserved in terms of the regulatory interactions between the signalling pathways and transcription factors that form its components (figure 9*c*). However, due to the drastically different shape and size of the limb bud in mice and sharks, the patterns that result from the process are radically different. This led to a fundamental reassessment of the evolutionary origin and homology of distal limb patterning systems: even though sharks do not have any morphological structures that even remotely resemble the tetrapod autopod, the underlying dynamical modules are highly conserved (see [14] for a discussion of process homology). It will be interesting to apply this kind of approach to other vertebrates with a diverse range of fore- and hind-limb morphologies. A shared underlying dynamical module may explain why they are all, in the end, variations of a common theme.

Dynamical modules also occur outside the animal kingdom. In plants, dynamical modules have been described in the regulatory systems that produce regularly spaced patterns of ‘hairs’ (trichomes) on the root and leaf epidermis [132,133] (reviewed in [134]). These modules are redundant in the sense that they are all sufficient but not necessary to produce the output pattern of the whole system. Consistent with our definition of dynamical modules, these subsystems all get to their final output in a different way. They have distinguishable activity-functions. While the functioning of each module in isolation is dependent on quite specific background conditions, they can generate their patterning output robustly when operating in concert. Here, individual activity-functions do not contribute different aspects to the correct output of the system. Instead, they are responsible for the robustness of patterning.

## 7. Conclusion

In this paper, we have presented a new account of dynamical modularity. We propose a top-down approach to systems decomposition, able to deal with the complex, feedback-driven dynamics characteristic of biological networks. It aims to identify and characterize dynamical modules as subprocesses within the overall behaviour of a regulatory system. Such subprocesses are defined through their activity-functions, which correspond to particular dynamical regimes. Put together, these regimes reproduce the robust dynamical repertoire of the whole system. We have demonstrated how dynamical modules can be detected algorithmically in discrete network models, while more heuristic and pragmatic methods are required for continuous cases. Finally, we have illustrated the wide applicability, the potential and some of the limitations of our approach through numerous examples drawn from metabolic, cellular and developmental regulation in bacteria, plants and animals.

We see the main domain of application for our account of dynamical modularity in cell and developmental biology. In addition, it also has important implications for evolution. The quasi-independence of phenotypic traits implies some kind of functional modularity in the epigenotype—the collection of generative processes that constitute the genotype–phenotype map (figure 1) [1,12,13,15–19,21,40,135]. Such functional dissociability is a prerequisite for evolvability [8]. By contrast, structural modularity is not. First of all, function is not determined by structure. Even simple networks can exhibit a range of different behaviours. Second, many regulatory networks with dissociable functions are not structurally modular [71]. In fact, structural integration may be a natural outcome of evolution by natural selection (see [38]; see also [1]). Therefore, an alternative approach is required to explain functional dissociability in networks that exhibit limited structural modularity.

Dynamical modules provide such an alternative approach. Unlike structure and function, activity-functions and use-functions are very closely related: what a subsystem *does* is also what it *contributes* to overall behaviour. Moreover, its dynamical repertoire is what determines the variational properties (and ultimately the evolvability) of an adaptive system. Probabilities of possible phenotypic transitions are governed by the geometry of configuration space [1,12,13,21]. To move from one dynamical regime to another, there must be a bifurcation set connecting them. In this way, the dynamical repertoire of a system defines its evolutionary space of possibilities. Since dynamical modules are subsystems characterized by distinct dynamical regimes, they will also have different evolutionary potentials. In other words, they will be variational modules, able to evolve quasi-independently.

This leads us to another important point: dynamical modules are subsystems with specific identifiable dynamical properties and some degree of independence from other modules. We have seen in §6 that they correspond to morphogenetic fields—individualized processes that can be localized in their spatio-temporal and regulatory context. In addition, they can vary quasi-independently during evolution. Taken together, these characteristics mean that dynamical modules are fundamental units both of ontogenesis and of evolution. As such, they provide the causal-mechanistic basis for a general theory of process homology [136]. This specific argument is elaborated further by DiFrisco & Jaeger [14]. More generally, dynamical modules provide a powerful framework for bridging the gap between structure and function across biological disciplines.

We are convinced that our dynamical approach to modularity will prove to be useful beyond the examples from cell, developmental and evolutionary biology presented here. Its potential for studies in cognitive neuroscience should be quite obvious. Cognitive processes can be subdivided into activity-functions that do not necessarily map to specific structures in the brain. Similarly, we see great potential for our approach in the social sciences, especially economics. The application of dynamical modularity beyond biology is currently limited by the fact that dynamical modules are more cryptic than structural or variational ones and require dynamical models—preferably validated by empirical, time-resolved data—for their detection and characterization [137,138]. Characterizing activity-functions through dynamical regimes requires us to explore the local structure of configuration space—the phase-space topologies accessible to the system. The relative arrangement of attractors and their basins, as well as the bifurcations that modify such arrangements, are what define the dynamic repertoire. A wider application of dynamical modularity would require either more studies based on data-driven dynamical modelling, or novel methods to characterize attractors and their bifurcation directly from sets of time-series data. It is still early days, and both of these prospects pose some truly daunting challenges. But they should be worth the effort if we consider the potential advances in understanding, advances that we hope to have illustrated in this paper.

## Data accessibility

This article has no additional data.
